# Crystal structure and Hirshfeld surface analysis of *N*-(4-nitro­phen­yl)-2-(piperidin-1-yl)acetamide (lidocaine analogue)

**DOI:** 10.1107/S205698902401185X

**Published:** 2025-01-01

**Authors:** Imane Maimoune, Benson M. Kariuki, Abderrazzak El Moutaouakil Ala Allah, Intissar Nchioua, Abdulsalam Alsubari, Joel T. Mague, Abdelkader Zarrouk, Youssef Ramli

**Affiliations:** a Laboratory of Materials Nanotechnology and Environment Faculty of Sciences Mohammed V University in Rabat PO Box 1014 Rabat Morocco; b Laboratory of Medicinal Chemistry, Drug Sciences Research Center, Faculty of Medicine and Pharmacy, Mohammed V University, Rabat, Morocco; c School of Chemistry, Cardiff University, Main Building, Park Place, Cardiff, CF10 3AT, United Kingdom; d Laboratory of Medicinal Chemistry, Faculty of Clinical Pharmacy, 21 September University, Yemen; e Department of Chemistry, Tulane University, New Orleans, LA 70118, USA; Katholieke Universiteit Leuven, Belgium

**Keywords:** crystal structure, acetamide, piperidine, hydrogen bond, C=O⋯π(ring) inter­action

## Abstract

In the title mol­ecule, C_13_H_17_N_3_O_3_, the substituents on the phenyl ring are rotated slightly out of the mean plane of the ring but the piperidine moiety is nearly perpendicular to that plane. In the crystal, C—H⋯O hydrogen bonds form chains of mol­ecules extending along the *c*-axis direction, which are linked by C=O⋯π(ring) inter­actions. A Hirshfeld surface analysis showed the majority of inter­molecular inter­actions to be H⋯H contacts while O⋯H/H⋯O contacts are the second most numerous.

## Abstract 2

The substituents on the phenyl ring are rotated slightly out of the mean plane of the ring while the piperidine moiety is nearly perpendicular to that plane. In the crystal, C—H⋯O hydrogen bonds form chains of mol­ecules extending along the *c*-axis direction, which are linked by C=O⋯π(ring) inter­actions. A Hirshfeld surface analysis showed the majority of inter­molecular inter­actions to be H⋯H contacts while O⋯H/H⋯O contacts are the second most numerous.

## 1. Chemical context

Heterocyclic compounds, especially those containing a nitro­gen atom, are of substantial inter­est in medicinal chemistry (El Moutaouakil Ala Allah *et al.*, 2024 ▸). Extensive studies of the acetamide family have demonstrated that it can be present in various known drugs of different classes with different thera­peutic activities (Rahim *et al.*, 2015 ▸; Bennani *et al.*, 2020 ▸; Karrouchi *et al.*, 2018 ▸). Their structural similarity to various bioactive natural and synthetic mol­ecules grants them a broad spectrum of biological activities (Ettahiri *et al.*, 2024 ▸). Lidocaine is a heterocyclic compound that acts as a local anesthetic (Calatayud & Gonzalez, 2003 ▸). It consists of a lipophilic aromatic ring and a hydro­philic amine. Its main target in excitable cells is the voltage-gated sodium channel, responsible for the increased sodium permeability observed during the rising phase of the action potential in peripheral nerves, skeletal muscles, as well as in neuroendocrine and cardiac cells (Costa *et al.*, 2008 ▸). Continuing our research in this area (Missioui *et al.*, 2022*b* ▸; Guerrab *et al.*, 2021 ▸; Mortada *et al.*, 2024 ▸) we synthesized the lidocaine analogue *N*-(4-nitro­phen­yl)-2-(piperidin-1-yl)acetamide through an alkyl­ation reaction of 2-chloro-*N*-(4-nitro­phen­yl)acetamide and piperidine, conducted under reflux in toluene. This paper presents the crystal structure of this lidocaine analogue, **3**. A Hirshfeld surface analysis was performed to analyze the inter­molecular inter­actions.
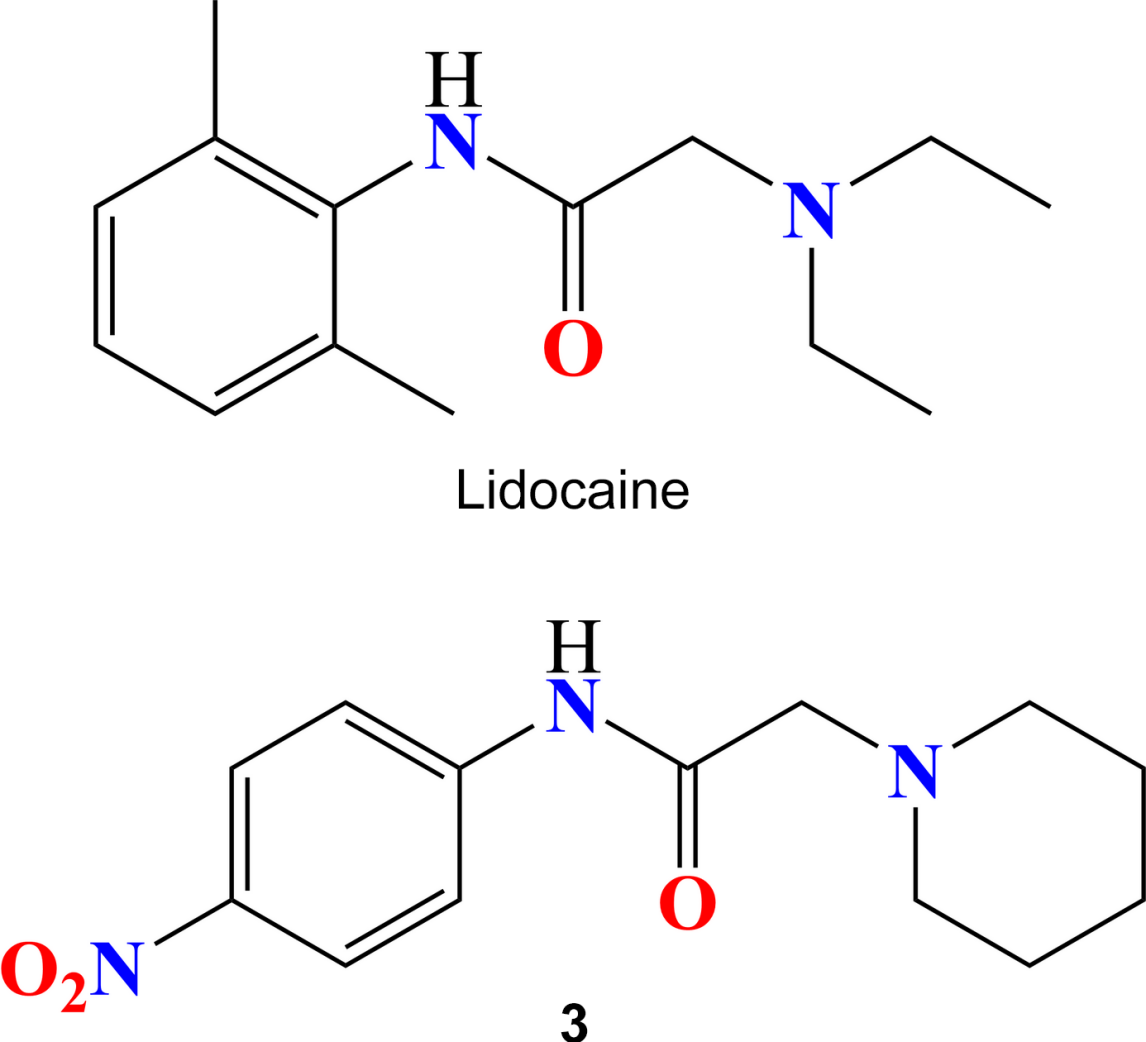


## 2. Structural commentary

**Figure 1:**
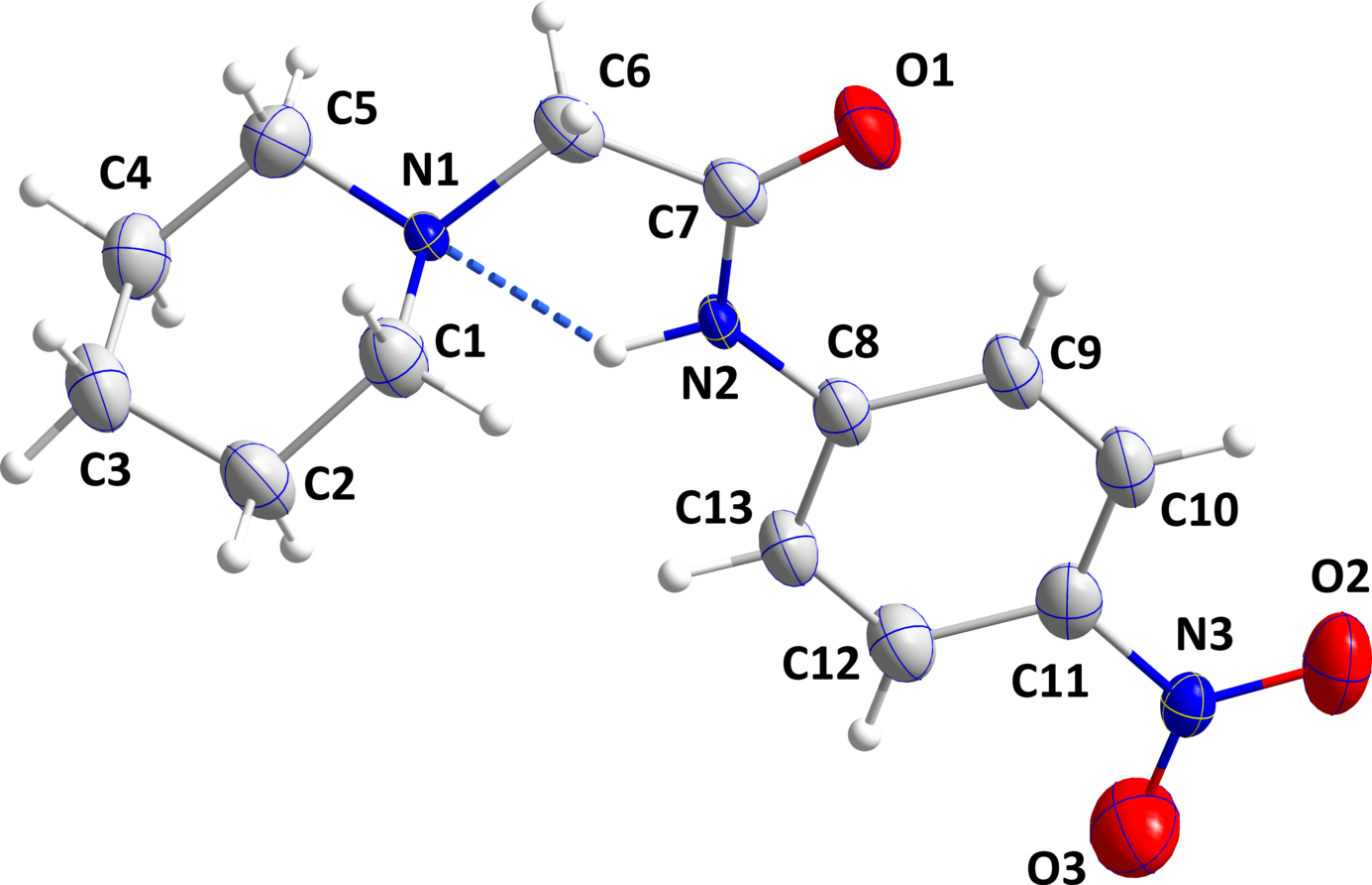
Perspective view of the title mol­ecule with labeling scheme and 30% probability ellipsoids. The intra­molecular hydrogen bond is depicted by a dashed line.

**Figure 2:**
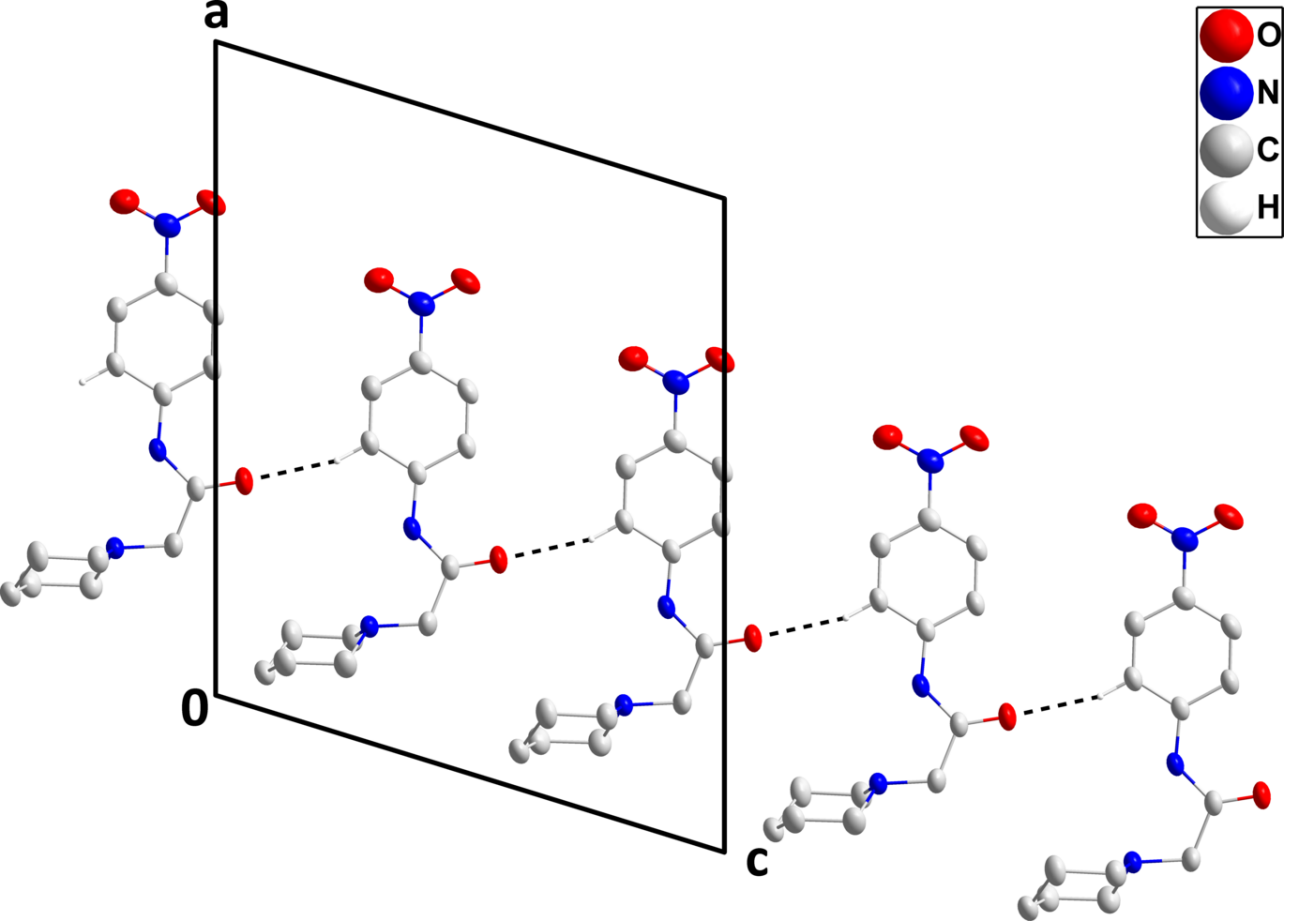
A portion of one chain viewed along the *b*-axis direction with the inter­molecular C—H⋯O hydrogen bonds depicted by black dashed lines. Hydrogen atoms not involved in these inter­actions are omitted for clarity.

The title compound, **3**, crystallizes in the monoclinic space group *P*2_1_/*c* with one mol­ecule in the asymmetric unit (Fig. 1 ▸). The nitro group is rotated 2.84 (3)° out of the mean plane of the attached phenyl ring while the dihedral angle between the plane defined by C8, N2, C7 and O1 and the mean plane of the phenyl ring is 4.52 (3)°. The C1—N1—C6—C7 torsion angle is 92.8 (2)°, which places the mean plane of the piperidine ring nearly perpendicular to the remainder of the mol­ecule, which is particularly evident in Figs. 2 ▸ and 4 and is partly due to the intra­molecular N2—H2⋯N1 hydrogen bond (Fig. 1 ▸ and Table 1 ▸). The piperidine ring adopts a chair conformation with puckering parameters (Cremer & Pople, 1975 ▸) *Q* = 0.559 (2) Å, θ = 178.1 (2)° and φ = 199 (8)°.

## 3. Supra­molecular features

**Figure 3:**
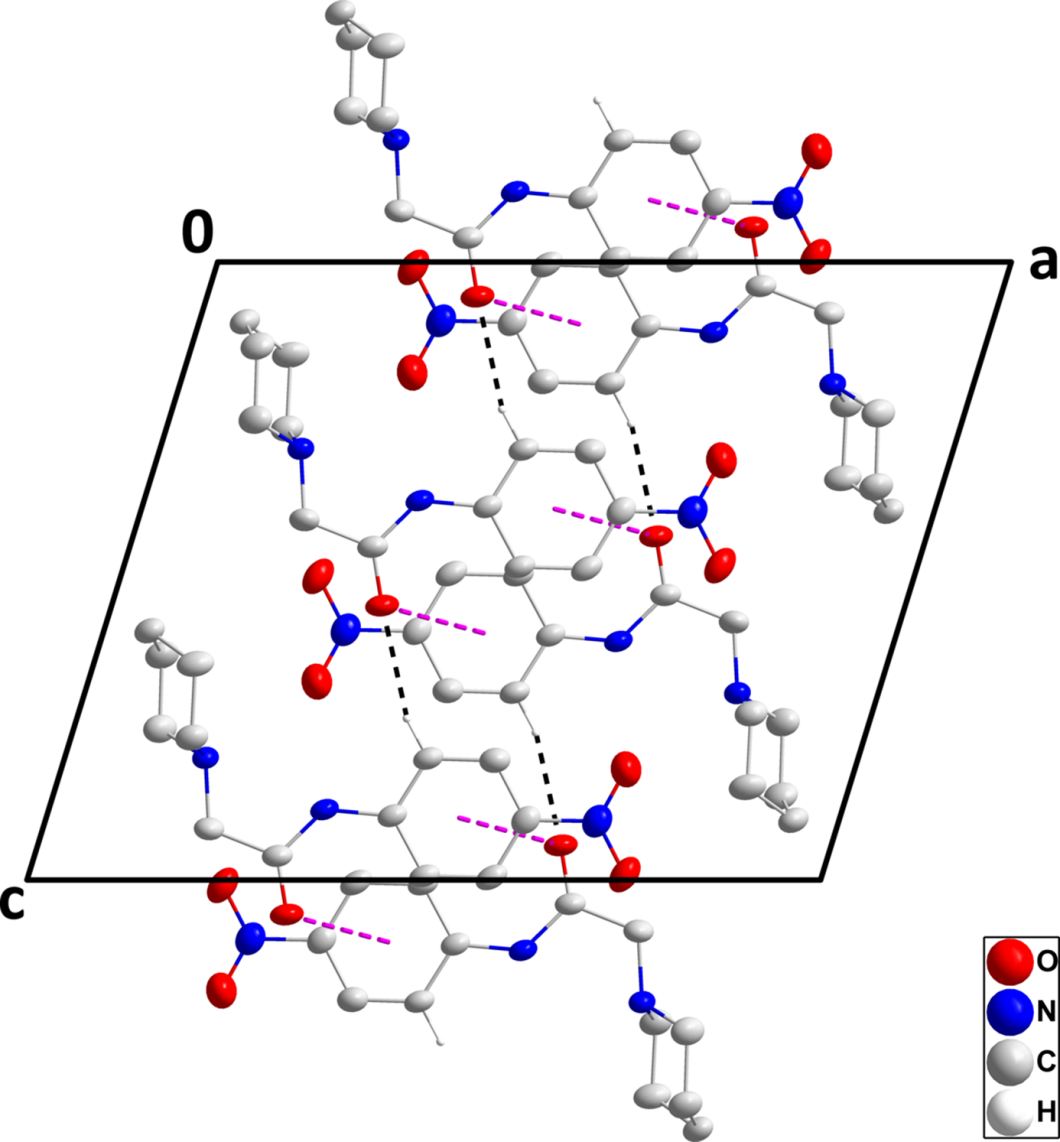
Packing viewed along the *b*-axis direction showing the pairing of two chains through C=O⋯π(ring) inter­actions (pink dashed lines). The C—H⋯O hydrogen bonds are depicted by black dashed lines. Hydrogen atoms not involved in these inter­actions are omitted for clarity.

In the crystal, C13—H13⋯O1^i^ hydrogen bonds (Table 1 ▸) form chains of mol­ecules extending along the *c*-axis direction (Fig. 2 ▸). These are linked in pairs across centers of symmetry by C7=O1⋯*Cg*2 inter­actions [*Cg*2 is the centroid of the C8–C13 ring at −*x* + 1, −*y* + 2, −*z* + 1; O1⋯*Cg*2 = 3.9066 (7) Å, C7⋯*Cg*2 = 4.274 (2), C7=O1⋯*Cg*2 = 99.16 (12)°] (Figs. 3 ▸ and 4 ▸).

## 4. Database survey

**Figure 5:**
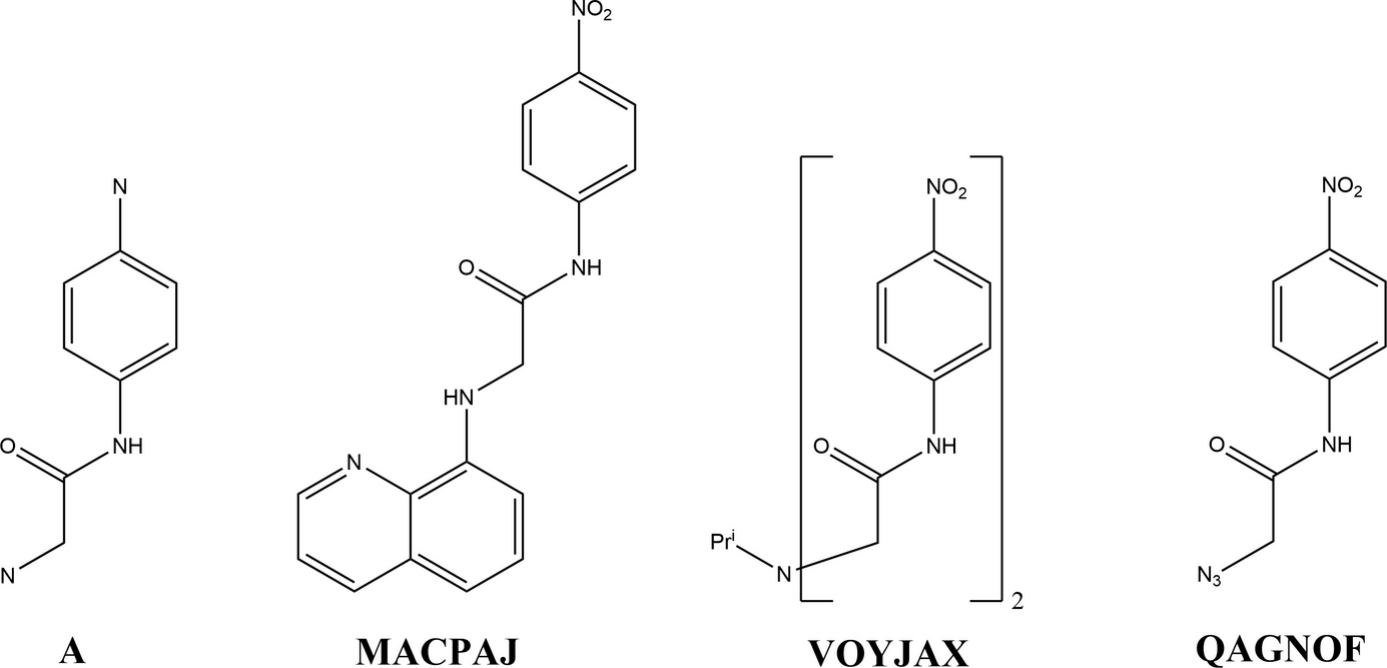
The search fragment used for the Database Survey (A) and three relevant hits generated.

A search of the Cambridge Structural Database (CSD, updated to June 2024 (Groom *et al.*, 2016 ▸)) with the search fragment **A** shown in Fig. 5 ▸ yielded eleven hits of which five had the fragment as part of multidentate ligands in metal complexes while three more were ionic compounds. None of these were considered relevant for comparison with the title mol­ecule. The three structures that are relevant are shown in Fig. 5 ▸.

In MACPAJ (Kang *et al.*, 2010 ▸), the rotation of the nitro group out of the plane of the attached phenyl ring is virtually the same as in the title mol­ecule, but the dihedral angle between the mean plane of the acetamido group and that of the phenyl ring is significantly greater at 13.80 (8)°. On the other hand, the entire mol­ecule is relatively flat as the mean planes of the phenyl and quinoline moieties are inclined to one another by 8.02 (7)°. The packing involves chains of alternating mol­ecules and water mol­ecules, which are formed by N—H⋯O and O—H⋯O plus O—H⋯N hydrogen bonds and are linked by π-stacking inter­actions between the phenyl and quinoline units.

In QAGNOF (Missioui *et al.*, 2020 ▸), the mean planes of the nitro and acetamide groups are inclined to that of the phenyl ring by 5.9 (5) and 14.8 (1)°, respectively. The 3-D structure of the crystal consists of corrugated layers parallel to (10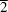), which are formed by C—H⋯O and C—H⋯N hydrogen bonds.

There is an intra­molecular N—H⋯O hydrogen bond in VOYJAX (Juraj *et al.*, 2019 ▸), which gives the mol­ecule a U-shaped conformation. The dihedral angles between the mean planes of the acetamido groups and their attached phenyl rings are both about 13°, while the nitro group on the portion containing the NH group that forms the intra­molecular hydrogen bond is rotated by 8.7° relative to the plane of its phenyl group, and the other nitro group is rotated by 5.6°. The other NH group forms inter­molecular N—H⋯O hydrogen bonds, generating chains extending along the normal to (201). These are connected into a 3-D network by a large number of C—H⋯O hydrogen bonds and offset π-stacking inter­actions.

## 5. Hirshfeld surface analysis

**Figure 6:**
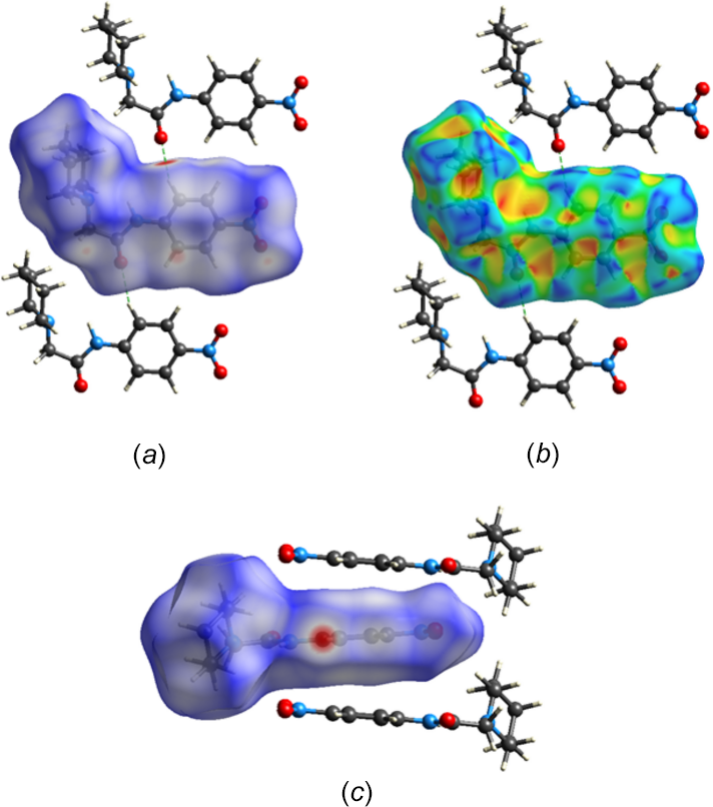
Hirshfeld surfaces: (*a*) *d*_norm_ viewed perpendicular to the plane of the phenyl ring with two adjacent hydrogen-bonded mol­ecules, (*b*) same view of the shape-index surface, (c) *d*_norm_ viewed parallel to the plane of the phenyl ring with two adjacent stacking mol­ecules.

**Figure 7:**
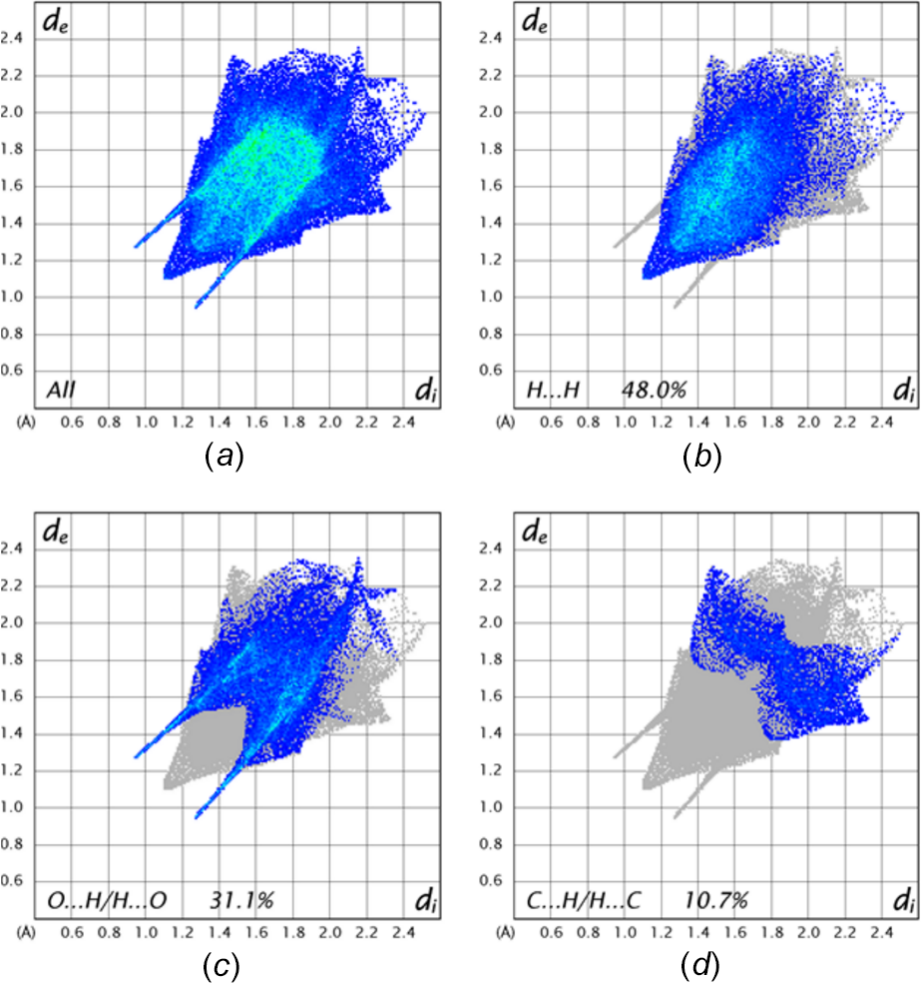
2-D fingerprint plots: (*a*) all inter­molecular inter­actions, and those delineated into (*b*) H⋯H, (*c*) O⋯H/H⋯O and (*d*) C⋯H/H⋯C inter­actions.

The Hirshfeld surface analysis was carried out with *CrystalExplorer* (Spackman *et al.*, 2021 ▸) and the descriptions and inter­pretations of the plots generated have been described previously (Tan *et al.*, 2019 ▸). The *d*_norm_ surface calculated over the range −0.2975 to 1.2755 in arbitrary units is shown in Fig. 6 ▸*a* and includes two neighboring mol­ecules attached *via* C—H⋯O hydrogen bonds, which are also indicated by the dark-red spots. This view corresponds to that in Fig. 2 ▸. The Hirshfeld surface calculated over the shape-index function is shown in Fig. 6 ▸*b* with the set of blue and orange triangles offset from the center of the benzene ring indicating the C=O⋯*Cg* inter­actions. Fig. 6 ▸*c* shows the *d*_norm_ surface viewed parallel to the plane of the benzene ring and includes two of the adjacent mol­ecules involved in the C=O⋯*Cg* stacking inter­actions. A representation of all inter­molecular inter­actions is given in Fig. 7 ▸*a* with delineations into H⋯H, O⋯H/H⋯O and C⋯H/H⋯C inter­actions, together with their percentage contributions, shown in Fig. 7 ▸*b*–7*d*, respectively. The high percentage attributed to H⋯H inter­actions is a consequence of the high hydrogen content of the mol­ecule and comes significantly from van der Waals contacts involving the piperidine moiety. Second and third in importance are the O⋯H/H⋯O and the C⋯H/H⋯C contacts with the former appearing as a pair of sharp spikes indicating a narrow range of H⋯O distances. Despite the presence of C=O⋯*Cg* inter­actions, the O⋯C/C⋯O contacts contribute only 2.9% to the total.

## 6. Synthesis and crystallization

**Figure 8:**
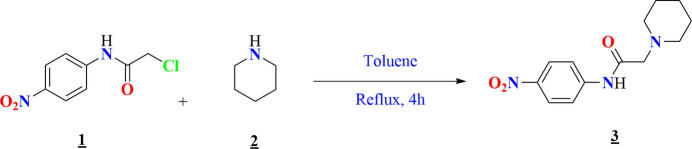
Reaction scheme for the formation of the title compound **3**.

The reaction sequence for title compound **3** is shown in Fig. 8 ▸.

Compound **1**, namely 2-chloro-*N*-(4-nitro­phen­yl)acetamide was synthesized according to the procedure described in the literature (Missioui *et al.*, 2022*a* ▸; Li *et al.*, 2006 ▸). Next, 1.2 mmol of piperidine **2** was mixed with **1** mmol of 2-chloro-*N*-(4-nitro­phen­yl)acetamide in toluene, and the mixture was refluxed for 4 h. Upon completion of the reaction, toluene was removed by liquid–liquid extraction, and the aqueous phase was subsequently acidified with hydro­chloric acid, prompting the precipitation of the title compound **3**. The precipitate was filtered, dried, and recrystallized from ethanol, yielding white crystals of the target compound.

Yield = 40%, color: white, m.p. = 401–403 K. FT–IR (ATR, ν, cm^−1^): 3214 (N—H amide), 2937 (C—H Aliphatic), 1692 (C=O). ^1^H NMR (500 MHz, DMSO-*d*_6_) δ ppm: 1.34 (*m*, 2H, C—CH_2_—C), 1.52 (*quint*, 4H, *J* = 5 Hz, C—CH_2_—C), 2.40 (*t*, 4H, *J* = 5 Hz, N—CH**_2_**–), 3.02 (*s*, 2H, CH**_2 amide_**), 7.05–7.65 (*m*, 4H, H—Ar), 9.67 (*s*, 1H, NH**_amide_**). ^13^C NMR (125 MHz, DMSO-*d*_6_) δ ppm: 25.10 (C—CH_2_—C), 24.01 (C—CH_2_—C), 53.85 (N—CH_2_—C), 61.48 (N—CH_2_—C=O), 128.80, 128.21, 135.50, 135.52 (C—Ar), 168.82 (C=O). HRMS (ESI): calculated for C_13_H_17_N_3_O_3_ [*M* + H]^+^ 263.1270; found 264.13318.

## 7. Refinement

Crystal data, data collection and structure refinement details are summarized in Table 2 ▸. Hydrogen atoms attached to carbon were included as riding contributions in idealized positions with isotropic displacement parameters tied to those of the attached atoms. That attached to nitro­gen N2 was located in a difference map and refined with a DFIX 0.91 0.01 instruction.

## Supplementary Material

Crystal structure: contains datablock(s) I. DOI: 10.1107/S205698902401185X/vm2309sup1.cif

Structure factors: contains datablock(s) I. DOI: 10.1107/S205698902401185X/vm2309Isup2.hkl

Supporting information file. DOI: 10.1107/S205698902401185X/vm2309Isup3.cml

CCDC reference: 2408229

Additional supporting information:  crystallographic information; 3D view; checkCIF report

## Figures and Tables

**Figure 4:**
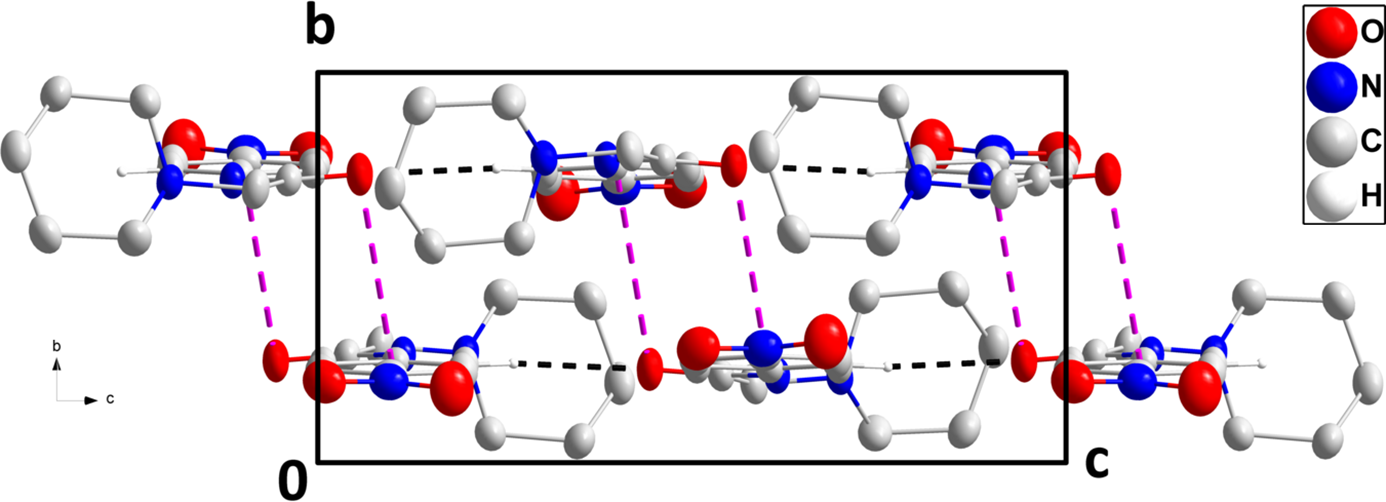
Packing viewed along the *a*-axis direction showing the pairing of two chains through C=O⋯π(ring) inter­actions (pink dashed lines). The C—H⋯O hydrogen bonds are depicted by black dashed lines. Hydrogen atoms not involved in these inter­actions are omitted for clarity.

**Table 1:** Hydrogen-bond geometry (Å, °)

*D*—H⋯*A*	*D*—H	H⋯*A*	*D*⋯*A*	*D*—H⋯*A*
N2—H2⋯N1	0.91 (1)	2.12 (2)	2.653 (2)	117 (2)
C13—H13⋯O1^i^	0.93	2.36	3.265 (2)	164

Symmetry code: (i) 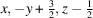.

**Table 2:** Experimental details

Crystal data	
Chemical formula	C_13_H_17_N_3_O_3_
*M*_r_	263.29
Crystal system, space group	Monoclinic, *P*2_1_/*c*
Temperature (K)	293
*a*, *b*, *c* (Å)	16.2304 (9), 6.5804 (3), 13.2222 (7)
β (°)	107.156 (6)
*V* (Å^3^)	1349.33 (13)
*Z*	4
Radiation type	Mo *K*α
μ (mm^−1^)	0.09
Crystal size (mm)	0.53 × 0.16 × 0.16

Data collection	
Diffractometer	SuperNova, Dual, Cu at home/near, Atlas
Absorption correction	Gaussian (CrysAlisPr; Rigaku OD, 2023 ▸)
*T*_min_, *T*_max_	0.564, 1.000
No. of measured, independent and observed [*I* > 2σ(*I*)] reflections	11819, 3351, 2275
*R*_int_	0.030
(sin θ/λ)_max_ (Å^−1^)	0.696

Refinement	
*R*[*F*^2^ > 2σ(*F*^2^)], *wR*(*F*^2^), *S*	0.059, 0.180, 1.06
No. of reflections	3351
No. of parameters	175
No. of restraints	1
H-atom treatment	H atoms treated by a mixture of independent and constrained refinement
Δρ_max_, Δρ_min_ (e Å^−3^)	0.14, −0.21

Computer programs: *CrysAlis PRO* (Rigaku OD, 2023 ▸), *SHELXT* (Sheldrick, 2015*a* ▸), *SHELXL* (Sheldrick, 2015*b* ▸), *DIAMOND* (Brandenburg & Putz, 2012 ▸) and *SHELXTL* (Sheldrick, 2008 ▸).

## supplementary crystallographic information

### N-(4-Nitrophenyl)-2-(piperidin-1-yl)acetamide Crystal data

**Table:**

C_13_H_17_N_3_O_3_	*F*(000) = 560
*M**_r_* = 263.29	*D*_x_ = 1.296 Mg m^−^^3^
Monoclinic, *P*2_1_/*c*	Mo *K*α radiation, λ = 0.71073 Å
*a* = 16.2304 (9) Å	Cell parameters from 4698 reflections
*b* = 6.5804 (3) Å	θ = 4.1–28.9°
*c* = 13.2222 (7) Å	µ = 0.09 mm^−^^1^
β = 107.156 (6)°	*T* = 293 K
*V* = 1349.33 (13) Å^3^	Block, colourless
*Z* = 4	0.53 × 0.16 × 0.16 mm

### N-(4-Nitrophenyl)-2-(piperidin-1-yl)acetamide Data collection

**Table:**

SuperNova, Dual, Cu at home/near, Atlas diffractometer	2275 reflections with *I* > 2σ(*I*)
Detector resolution: 10.5082 pixels mm^-1^	*R*_int_ = 0.030
ω scans	θ_max_ = 29.7°, θ_min_ = 3.4°
Absorption correction: gaussian (CrysAlisPr; Rigaku OD, 2023)	*h* = −22→17
*T*_min_ = 0.564, *T*_max_ = 1.000	*k* = −8→9
11819 measured reflections	*l* = −13→18
3351 independent reflections	

### N-(4-Nitrophenyl)-2-(piperidin-1-yl)acetamide Refinement

**Table:**

Refinement on *F*^2^	Primary atom site location: dual
Least-squares matrix: full	Secondary atom site location: difference Fourier map
*R*[*F*^2^ > 2σ(*F*^2^)] = 0.059	Hydrogen site location: mixed
*wR*(*F*^2^) = 0.180	H atoms treated by a mixture of independent and constrained refinement
*S* = 1.06	*w* = 1/[σ^2^(*F*_o_^2^) + (0.0632*P*)^2^ + 0.563*P*] where *P* = (*F*_o_^2^ + 2*F*_c_^2^)/3
3351 reflections	(Δ/σ)_max_ < 0.001
175 parameters	Δρ_max_ = 0.14 e Å^−^^3^
1 restraint	Δρ_min_ = −0.21 e Å^−^^3^

### N-(4-Nitrophenyl)-2-(piperidin-1-yl)acetamide Special details

**Table:**

Geometry. All esds (except the esd in the dihedral angle between two l.s. planes) are estimated using the full covariance matrix. The cell esds are taken into account individually in the estimation of esds in distances, angles and torsion angles; correlations between esds in cell parameters are only used when they are defined by crystal symmetry. An approximate (isotropic) treatment of cell esds is used for estimating esds involving l.s. planes.
Refinement. Refinement of F^2^ against ALL reflections. The weighted R-factor wR and goodness of fit S are based on F^2^, conventional R-factors R are based on F, with F set to zero for negative F^2^. The threshold expression of F^2^ > 2sigma(F^2^) is used only for calculating R-factors(gt) etc. and is not relevant to the choice of reflections for refinement. R-factors based on F^2^ are statistically about twice as large as those based on F, and R- factors based on ALL data will be even larger. H-atoms attached to carbon were placed in calculated positions (C—H = 0.95 - 0.98 Å) and were included as riding contributions with isotropic displacement parameters 1.2 - 1.5 times those of the attached atoms. That attached to nitrogen was placed in a location derived from a difference map and refined with a DFIX 0.91 0.01 instruction.

### N-(4-Nitrophenyl)-2-(piperidin-1-yl)acetamide Fractional atomic coordinates and isotropic or equivalent isotropic displacement parameters (Å^2^)

**Table:**

	*x*	*y*	*z*	*U*_iso_*/*U*_eq_	
O1	0.34083 (11)	0.7609 (2)	0.55602 (11)	0.0715 (5)	
O2	0.75115 (11)	0.7076 (3)	0.49135 (16)	0.0883 (6)	
O3	0.71190 (13)	0.6897 (4)	0.32098 (19)	0.1075 (7)	
N1	0.17750 (10)	0.7809 (2)	0.30235 (12)	0.0514 (4)	
N2	0.34754 (11)	0.7767 (3)	0.38608 (12)	0.0544 (4)	
H2	0.3113 (14)	0.794 (4)	0.3199 (11)	0.082*	
N3	0.69567 (13)	0.7073 (3)	0.40483 (19)	0.0725 (6)	
C1	0.15223 (15)	0.5745 (3)	0.26946 (15)	0.0637 (6)	
H1A	0.103397	0.535977	0.293408	0.076*	
H1B	0.199494	0.482909	0.301768	0.076*	
C2	0.12820 (17)	0.5550 (4)	0.15026 (17)	0.0738 (7)	
H2A	0.178609	0.580913	0.127050	0.089*	
H2B	0.108885	0.417421	0.129819	0.089*	
C3	0.05749 (15)	0.7031 (4)	0.09660 (17)	0.0699 (6)	
H3A	0.047410	0.698021	0.020587	0.084*	
H3B	0.004423	0.665403	0.111393	0.084*	
C4	0.08291 (16)	0.9145 (4)	0.13599 (17)	0.0726 (7)	
H4A	0.034863	1.005917	0.106948	0.087*	
H4B	0.130725	0.959234	0.111583	0.087*	
C5	0.10881 (16)	0.9242 (4)	0.25522 (17)	0.0706 (6)	
H5A	0.128024	1.060726	0.278232	0.085*	
H5B	0.059199	0.893836	0.279295	0.085*	
C6	0.20950 (14)	0.7987 (4)	0.41715 (15)	0.0635 (6)	
H6A	0.181932	0.695836	0.448581	0.076*	
H6B	0.193088	0.930527	0.437812	0.076*	
C7	0.30609 (14)	0.7755 (3)	0.46103 (14)	0.0539 (5)	
C8	0.43526 (12)	0.7593 (3)	0.39598 (14)	0.0484 (4)	
C9	0.49923 (14)	0.7525 (3)	0.49329 (16)	0.0564 (5)	
H9	0.484448	0.759105	0.556019	0.068*	
C10	0.58435 (14)	0.7361 (3)	0.49582 (17)	0.0597 (5)	
H10	0.627409	0.731472	0.560310	0.072*	
C11	0.60535 (13)	0.7267 (3)	0.40304 (17)	0.0561 (5)	
C12	0.54306 (15)	0.7333 (3)	0.30645 (17)	0.0648 (6)	
H12	0.558375	0.726652	0.244073	0.078*	
C13	0.45840 (14)	0.7497 (3)	0.30335 (15)	0.0588 (5)	
H13	0.415929	0.754475	0.238376	0.071*	

### N-(4-Nitrophenyl)-2-(piperidin-1-yl)acetamide Atomic displacement parameters (Å^2^)

**Table:**

	*U*^11^	*U*^22^	*U*^33^	*U*^12^	*U*^13^	*U*^23^
O1	0.0773 (10)	0.0929 (12)	0.0357 (7)	−0.0018 (8)	0.0035 (7)	0.0013 (7)
O2	0.0574 (9)	0.0966 (13)	0.0938 (14)	−0.0027 (8)	−0.0041 (9)	0.0062 (10)
O3	0.0747 (12)	0.155 (2)	0.0976 (16)	0.0010 (12)	0.0324 (11)	0.0016 (14)
N1	0.0505 (8)	0.0634 (10)	0.0369 (8)	0.0029 (7)	0.0078 (6)	−0.0033 (7)
N2	0.0536 (9)	0.0673 (10)	0.0343 (8)	0.0003 (8)	0.0008 (7)	0.0000 (7)
N3	0.0610 (11)	0.0701 (12)	0.0798 (14)	−0.0034 (9)	0.0108 (10)	0.0025 (10)
C1	0.0731 (14)	0.0621 (13)	0.0494 (11)	−0.0004 (10)	0.0079 (10)	0.0051 (9)
C2	0.0935 (17)	0.0671 (14)	0.0507 (12)	−0.0034 (12)	0.0053 (11)	−0.0092 (10)
C3	0.0600 (12)	0.0934 (17)	0.0477 (11)	−0.0108 (12)	0.0026 (9)	0.0027 (11)
C4	0.0769 (15)	0.0764 (15)	0.0562 (12)	0.0126 (12)	0.0067 (11)	0.0133 (11)
C5	0.0784 (15)	0.0684 (14)	0.0596 (13)	0.0169 (11)	0.0122 (11)	−0.0009 (11)
C6	0.0672 (13)	0.0820 (15)	0.0397 (10)	0.0038 (11)	0.0132 (9)	−0.0078 (10)
C7	0.0652 (12)	0.0547 (11)	0.0364 (9)	−0.0022 (9)	0.0067 (8)	−0.0030 (8)
C8	0.0550 (11)	0.0428 (9)	0.0391 (9)	−0.0023 (8)	0.0012 (8)	−0.0008 (7)
C9	0.0601 (11)	0.0617 (12)	0.0380 (9)	−0.0029 (9)	−0.0001 (8)	0.0002 (8)
C10	0.0597 (12)	0.0561 (11)	0.0491 (11)	−0.0030 (9)	−0.0058 (9)	0.0015 (9)
C11	0.0557 (11)	0.0460 (10)	0.0590 (12)	−0.0027 (8)	0.0052 (9)	0.0020 (8)
C12	0.0682 (14)	0.0749 (14)	0.0471 (11)	−0.0011 (11)	0.0105 (10)	0.0012 (10)
C13	0.0589 (12)	0.0707 (13)	0.0383 (9)	0.0004 (9)	0.0010 (8)	−0.0001 (9)

### N-(4-Nitrophenyl)-2-(piperidin-1-yl)acetamide Geometric parameters (Å, º)

**Table:**

O1—C7	1.219 (2)	C4—C5	1.508 (3)
O2—N3	1.230 (3)	C4—H4A	0.9700
O3—N3	1.218 (3)	C4—H4B	0.9700
N1—C1	1.448 (3)	C5—H5A	0.9700
N1—C5	1.453 (3)	C5—H5B	0.9700
N1—C6	1.457 (2)	C6—C7	1.510 (3)
N2—C7	1.352 (3)	C6—H6A	0.9700
N2—C8	1.396 (3)	C6—H6B	0.9700
N2—H2	0.906 (10)	C8—C13	1.384 (3)
N3—C11	1.465 (3)	C8—C9	1.395 (2)
C1—C2	1.513 (3)	C9—C10	1.376 (3)
C1—H1A	0.9700	C9—H9	0.9300
C1—H1B	0.9700	C10—C11	1.368 (3)
C2—C3	1.513 (3)	C10—H10	0.9300
C2—H2A	0.9700	C11—C12	1.376 (3)
C2—H2B	0.9700	C12—C13	1.367 (3)
C3—C4	1.500 (3)	C12—H12	0.9300
C3—H3A	0.9700	C13—H13	0.9300
C3—H3B	0.9700		
			
C1—N1—C5	111.50 (17)	N1—C5—C4	111.27 (18)
C1—N1—C6	111.77 (17)	N1—C5—H5A	109.4
C5—N1—C6	112.78 (16)	C4—C5—H5A	109.4
C7—N2—C8	130.18 (16)	N1—C5—H5B	109.4
C7—N2—H2	112.8 (17)	C4—C5—H5B	109.4
C8—N2—H2	117.0 (17)	H5A—C5—H5B	108.0
O3—N3—O2	123.5 (2)	N1—C6—C7	113.75 (17)
O3—N3—C11	118.5 (2)	N1—C6—H6A	108.8
O2—N3—C11	118.0 (2)	C7—C6—H6A	108.8
N1—C1—C2	110.83 (17)	N1—C6—H6B	108.8
N1—C1—H1A	109.5	C7—C6—H6B	108.8
C2—C1—H1A	109.5	H6A—C6—H6B	107.7
N1—C1—H1B	109.5	O1—C7—N2	125.2 (2)
C2—C1—H1B	109.5	O1—C7—C6	121.1 (2)
H1A—C1—H1B	108.1	N2—C7—C6	113.72 (16)
C3—C2—C1	111.1 (2)	C13—C8—C9	119.5 (2)
C3—C2—H2A	109.4	C13—C8—N2	117.15 (16)
C1—C2—H2A	109.4	C9—C8—N2	123.38 (19)
C3—C2—H2B	109.4	C10—C9—C8	119.6 (2)
C1—C2—H2B	109.4	C10—C9—H9	120.2
H2A—C2—H2B	108.0	C8—C9—H9	120.2
C4—C3—C2	109.97 (18)	C11—C10—C9	119.71 (18)
C4—C3—H3A	109.7	C11—C10—H10	120.1
C2—C3—H3A	109.7	C9—C10—H10	120.1
C4—C3—H3B	109.7	C10—C11—C12	121.4 (2)
C2—C3—H3B	109.7	C10—C11—N3	120.15 (19)
H3A—C3—H3B	108.2	C12—C11—N3	118.4 (2)
C3—C4—C5	111.44 (19)	C13—C12—C11	119.2 (2)
C3—C4—H4A	109.3	C13—C12—H12	120.4
C5—C4—H4A	109.3	C11—C12—H12	120.4
C3—C4—H4B	109.3	C12—C13—C8	120.65 (18)
C5—C4—H4B	109.3	C12—C13—H13	119.7
H4A—C4—H4B	108.0	C8—C13—H13	119.7
			
C5—N1—C1—C2	58.7 (2)	C7—N2—C8—C9	−5.4 (3)
C6—N1—C1—C2	−174.01 (19)	C13—C8—C9—C10	−0.1 (3)
N1—C1—C2—C3	−56.4 (3)	N2—C8—C9—C10	−179.68 (18)
C1—C2—C3—C4	53.3 (3)	C8—C9—C10—C11	0.0 (3)
C2—C3—C4—C5	−53.0 (3)	C9—C10—C11—C12	0.0 (3)
C1—N1—C5—C4	−58.5 (3)	C9—C10—C11—N3	−179.44 (18)
C6—N1—C5—C4	174.8 (2)	O3—N3—C11—C10	176.6 (2)
C3—C4—C5—N1	55.8 (3)	O2—N3—C11—C10	−2.7 (3)
C1—N1—C6—C7	92.8 (2)	O3—N3—C11—C12	−2.9 (3)
C5—N1—C6—C7	−140.7 (2)	O2—N3—C11—C12	177.9 (2)
C8—N2—C7—O1	1.2 (3)	C10—C11—C12—C13	0.0 (3)
C8—N2—C7—C6	179.99 (19)	N3—C11—C12—C13	179.48 (19)
N1—C6—C7—O1	−170.56 (18)	C11—C12—C13—C8	−0.1 (3)
N1—C6—C7—N2	10.6 (3)	C9—C8—C13—C12	0.2 (3)
C7—N2—C8—C13	175.02 (19)	N2—C8—C13—C12	179.75 (19)

### N-(4-Nitrophenyl)-2-(piperidin-1-yl)acetamide Hydrogen-bond geometry (Å, º)

**Table:**

*D*—H···*A*	*D*—H	H···*A*	*D*···*A*	*D*—H···*A*
N2—H2···N1	0.91 (1)	2.12 (2)	2.653 (2)	117 (2)
C13—H13···O1^i^	0.93	2.36	3.265 (2)	164

Symmetry code: (i) *x*, −*y*+3/2, *z*−1/2.
